# Opposite effects of Gα_i2_ or Gα_i3_ deficiency on reduced basal density and attenuated β-adrenergic response of ventricular Ca^2+^ currents in myocytes of mice overexpressing the cardiac β_1_-adrenoceptor

**DOI:** 10.1007/s00210-025-03999-y

**Published:** 2025-03-31

**Authors:** Nour Katnahji, Jan Matthes

**Affiliations:** Center of Pharmacology, Department II, University of Cologne and University Hospital Cologne, Gleueler Strasse 24, Cologne, 50931 Germany

**Keywords:** Calcium channel, Adrenergic receptor, Inhibitory g protein GI, Heart failure, Cardiomyopathy, Transgenic mouse

## Abstract

Ca^2+^ currents (*I*_CaL_) carried by ventricular L-type Ca^2+^ channels (LTCC) are altered in failing hearts, and increased LTCC activity is discussed as a cause of cardiomyopathy. We have shown that lack of the inhibitory G-protein isoform Gα_i3_ improves cardiac outcome and survival in a murine heart-failure model of cardiac β_1_-adrenoceptor (β_1_-AR) overexpression (β_1_-tg), while lack of the Gα_i2_ isoform was detrimental in the same heart-failure model. Given the potential role of LTCC and their modulation by β-adrenergic signalling, we now analysed ventricular *I*_CaL_ in β_1_-tg mice and in β_1_-tg mice lacking either Gα_i2_ or Gα_i3_. Using the patch-clamp technique, we recorded whole-cell *I*_CaL_ in ventricular myocytes freshly isolated from adult mice. Compared to age-matched wild-type littermates, basal *I*_CaL_ was reduced in myocytes from β_1_-tg mice both under basal conditions (− 8.1 ± 1.6 vs. − 5.5 ± 1.5 pA/pF) and upon β-adrenergic stimulation with 1 µM isoproterenol (− 14.3 ± 5.6 vs. − 7.4 ± 1.9 pA/pF). Lack of Gα_i3_ normalised basal *I*_CaL_ to nearly wild-type levels (− 7.5 ± 1.6 pA/pF), while β-adrenergic response remained attenuated (− 9.5 ± 3.6 pA/pF). In contrast, the absence of Gα_i2_ did not restore basal *I*_CaL_ (− 5.7 ± 1.8 pA/pF), but restored the β-adrenergic response of *I*_CaL_, with the difference from basal current even exceeding that in wild-type mice (− 12.2 ± 2.9 pA/pF).We propose that by restoring basal *I*_CaL_, Gα_i3_ deficiency might contribute to the restoration of contractility in β_1_-tg mice, while maintaining attenuation of the *I*_CaL_ response upon β-adrenergic stimulation protects against deleterious effects mediated by enhanced β-AR signalling. In contrast, restored and even enhanced *I*_CaL_ response to β-adrenergic stimulation might contribute to detrimental effects of Gα_i2_ deficiency observed in β_1_-tg mice previously.

**Supplementary Information:**

The online version contains supplementary material available at 10.1007/s00210-025-03999-y.

## Introduction

Alterations of ventricular L-type Ca^2+^ currents (*I*_CaL_) have been associated with cardiomyopathy and heart failure in animal models and humans (Mukherjee and Spinale 1998; Richard et al. 1998; Schröder et al. 1998; Chen et al. 2002, 2008; Nakayama et al. 2007; Beetz et al. 2009). β-adrenoceptor (β-AR) overexpression and lack of Gα_i_ isoforms play a role both in the modulation of ventricular *I*_CaL_ and the development of cardiomyopathy (Engelhardt et al. 1999; Liggett et al. 2000; Foerster et al. 2003, 2004; Keller et al. 2015; Schröper et al. 2024). In the murine heart-failure model of β_1_-AR overexpression (β_1_-tg) (Engelhardt et al. 1999), an additional lack of Gα_i2_ (Gα_i2_^−/−^) led to early-onset heart failure in mice with cardiac overexpression of β_1_-AR (Keller et al. 2015). In contrast, we recently found that the heart-failure phenotype of β_1_-tg mice is prevented or at least delayed by additional Gα_i3_ deficiency (Schröper et al. 2024). Given the link between ventricular *I*_CaL_ and cardiac (dys-)function, we performed whole-cell *I*_CaL_ recordings using ventricular myocytes isolated from β_1_-tg mice and β_1_-tg mice lacking either Gα_i2_ or Gα_i3_ with an explorative intention. Our results revealed differences in ventricular *I*_CaL_ under basal conditions and upon β-adrenergic stimulation, which hint towards mechanisms underlying isoform-specific roles of Gα_i_ proteins.

## Material and methods

For details, please refer to supplementary data.

On a C57BL/6J background, mice with cardiac overexpression of the human β_1_-AR (Keller et al. 2015) were crossbred with mice globally lacking either Gα_i2_ (Dizayee et al. 2011) or Gα_i3_ (Gohla et al. 2007). Male mice at an age of 4–5 (β_1_-tg/Gα_i2_^−/−^) or 10–11 months (β_1_-tg/Gα_i3_^−/−^) were investigated. Age-matched wild-type and β_1_-tg mice were used for comparison. The federal state authority approved animal breeding, maintenance and experiments (references: 84-02.04.2016.A422 and 81-02.04.2022.A141). All animal experiments complied with the guidelines from Directive 2010/63/EU of the European Parliament on the protection of animals used for scientific purposes.

Ventricular myocytes were isolated by retrograde perfusion of freshly excised hearts with collagenase-containing solutions, kept at room temperature and subjected to patch-clamp experiments within 2–8 h.

By patch-clamp technique, we recorded ventricular whole-cell *I*_CaL_. Pipette solution (mM): 120 CsCl, 10 EGTA, 4 Mg-ATP, 5 HEPES, 1 MgCl_2_; pH 7.2. Bath solution (mM): 137 NaCl, 10 HEPES, 10 glucose, 5.4 CsCl, 2 CaCl_2_, 1 MgCl_2_; pH 7.4. I-V curves were obtained at room temperature using a double-pulse protocol. To correct for different cell size, *I*_CaL_ density was analysed, i.e. peak *I*_CaL_ divided by membrane capacitance (that was similar in all groups). For analysing voltage dependence of activation, data were fitted by combined Ohm and Boltzmann relation using $\text{I}\left(\text{V}\right)= \left(\text{V}-\text{ VR}\right)\times \frac{{G}_{max}}{(1+\text{exp}\frac{({V}_{0.5}-V)}{dV})}$ (Dizayee et al. 2011; Despang et al. 2022). We estimated the half-maximum potential of inactivation from steady-state inactivation curves by fitting with a sigmoidal Boltzmann equation, too (Poomvanicha et al. 2011). In addition to basal conditions, patch-clamp recordings were separately performed using cells incubated with 1 µM isoproterenol for 8–10 min.

Throughout, we present mean values ± standard deviation. More than two groups were compared using one-way ANOVA followed by Bonferroni-corrected post-tests. Two groups were compared using unpaired Student’s *t* test or the Mann-Whitney *U* test as appropriate. We considered *p* values < 0.05 statistically significant.

## Results

### Impaired ventricular I_CaL_ in β_1_-tg compared to WT cardiomyocytes

**Fig. 1:**
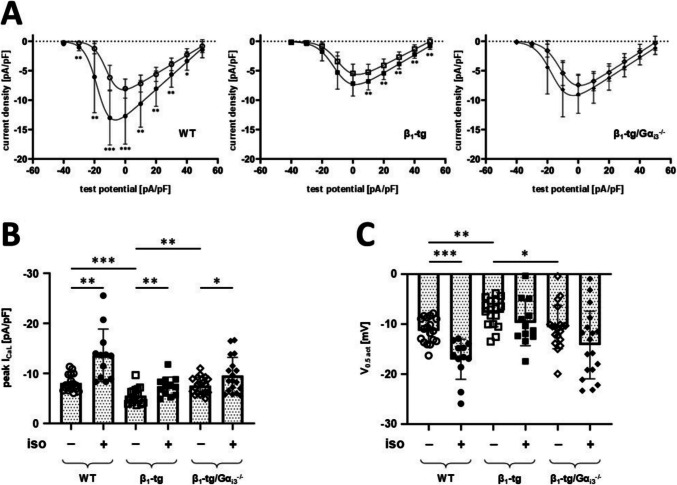
In β_1_-tg mice, lack of Gα_i3_ reverses the reduction in basal ventricular *I*_CaL_ but does not restore the response to β-adrenergic stimulation. Current-voltage relationships (**A**) show that both the increase of *I*_CaL_ density and the shift of activation upon β-adrenergic stimulation (closed symbols) are impaired in mice overexpressing the cardiac β_1_-adrenoceptor (β_1_-tg). Lack of Gα_i3_ (Gα_i3_^−/−^) does not reverse this effect. **B** Effect of isoproterenol (iso) on peak *I*_CaL_ density and **C** on the half-maximum potential of *I*_CaL_ activation. Data are presented as mean ± SD. Symbols in **B** and **C** represent values derived from individual recordings. *p < 0.05; **p < 0.01 and ***p < 0.001 in multiple unpaired *t* tests (**A**), unpaired *t* tests comparing effects of isoproterenol (**B**, **C**), or Bonferroni-corrected post-tests following one-way ANOVA from comparison of genotypes under basal conditions (**B**, **C**). To test β-adrenergic stimulation, cells were incubated with 1 µM isoproterenol for 8 ± 2 min. Data were obtained in *n* = 12–19 recordings with cells from at least three animals per genotype, aged 10–11 months

In β_1_-tg mice (10–11 months of age), ventricular peak *I*_CaL_ density was significantly reduced compared to age-matched WT, and *I*_CaL_ activation was shifted to more positive potentials (Fig. 1; Table S1). *I*_CaL_ response (peak *I*_CaL_ density and activation potential) to 1 µM isoproterenol was significantly reduced compared to WT. Thus, β_1_-tg mice showed altered ventricular *I*_CaL_ both under basal conditions and upon β-adrenergic stimulation at an age, when neither cardiac hypertrophy nor cardiac dysfunction were found in previous studies (Keller et al. 2015; Schröper et al. 2024).

### Additional Gα_i3_ deficiency normalizes basal I_CaL_ but not the response to isoproterenol in β_1_-tg cardiomyocytes

*I*_CaL_ density in β_1_-tg/Gα_i3_^−/−^ mice was higher than in β_1_-tg mice, and not significantly different from WT (Fig. 1; Table S1). There was no right-shift of the activation potential as in β_1_-tg mice. Overlap of *I*_CaL_ activation and inactivation curves suggests only slight differences with respect to window currents, i.e. currents flowing in a voltage range where inactivation is not yet complete while activation already occurs (Fig. S1). The blunted response to β-adrenergic stimulation mainly persisted in β_1_-tg/Gα_i3_^−/−^ mice. In contrast to β_1_-tg mice, however, isoproterenol shifted *I*_CaL_ inactivation significantly to more negative potentials. Taken together, Gα_i3_ deficiency led to normalization of ventricular *I*_CaL_ density and activation potential in β_1_-tg mice under basal conditions. Response to β-adrenergic stimulation, however, remained disturbed.

### Gα_i2_ deficiency does not restore basal I_CaL_ but response to isoproterenol in β_1_-tg cardiomyocytes

**Fig. 2:**
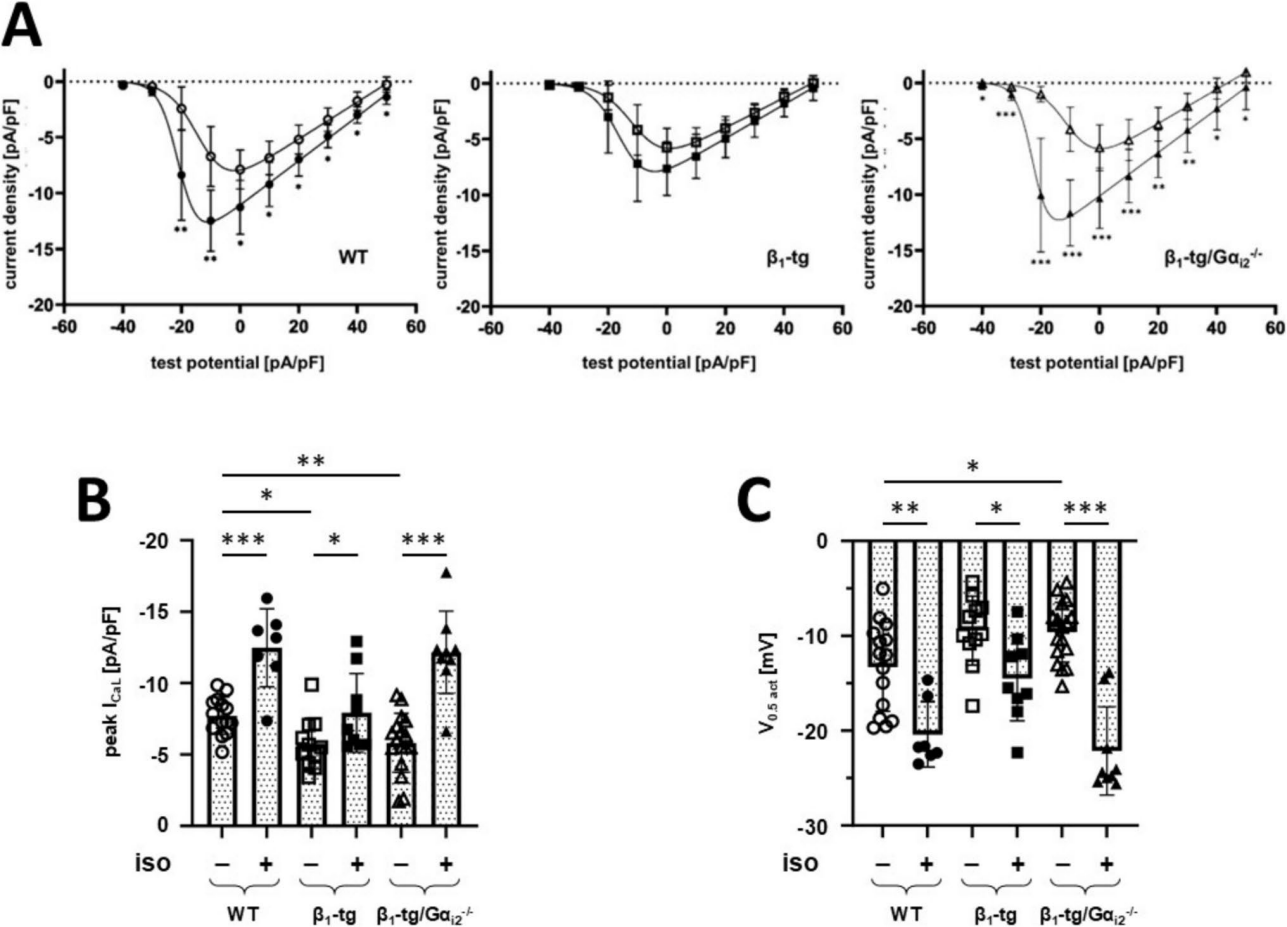
In β_1_-tg mice, lack of Gα_i2_ restores the β-adrenergic response without influencing the reduced basal *I*_CaL_. Current-voltage relationships (**A**) indicate a blunted increase of the *I*_CaL_ density upon β-adrenergic stimulation (closed symbols) by isoproterenol (iso) in mice overexpressing the cardiac β_1_-adrenoceptor (β_1_-tg), which is reversed in β_1_-tg mice lacking Gα_i2_ (β_1_-tg/Gα_i2_^−/−^). **B** Respective effects on peak *I*_CaL_ density and **C** on the half-maximum potential of *I*_CaL_ activation. Data are presented as mean ± SD. Symbols in **B** and **C** represent values derived from individual recordings. *p < 0.05; **p < 0.01 and ***p < 0.001 in multiple unpaired *t* tests (**A**), unpaired *t* tests comparing effects of isoproterenol (**B**, **C**), or Bonferroni-corrected post-tests following one-way ANOVA from comparison of genotypes under basal conditions (**B**, **C**). To test β-adrenergic stimulation, cells were incubated with 1 µM isoproterenol for 8 ± 2 min. Data were obtained in *n* = 7–17 recordings with cells from at least three animals per genotype, aged 4–5 months. WT, wild-type littermates

Our current findings on β_1_-tg mice aged 10–11 months suggest that changes in ventricular *I*_CaL_ precede contractile dysfunction previously observed at about 18 months (Schröper et al. 2024), while in another study, Gα_i2_ deficiency in β_1_-tg mice led to heart failure already at 10–11 months of age (Keller et al. 2015). Thus, we investigated *I*_CaL_ here at an even younger age of 4–5 months. Already at this younger age, peak *I*_CaL_ density was significantly reduced in β_1_-tg mice under basal conditions, and activation appeared to occur at more positive potentials (Fig. 2; Table S2). Furthermore, *I*_CaL_ response to isoproterenol incubation was attenuated. Basal *I*_CaL_ density and activation potentials were not normalized by Gα_i2_ deficiency, while inactivation was significantly shifted towards more positive potentials (Fig. S2). Overlapping curves of *I*_CaL_ activation and inactivation indicated an increased window current in case of β_1_-tg/Gα_i2_^−/−^ compared to both wild-type and β_1_-tg mice, respectively. *I*_CaL_ response to isoproterenol was at least restored in β_1_-tg/Gα_i2_^−/−^ mice. Isoproterenol caused a statistically significant leftward shift of the *I*_CaL_ activation potential in all three genotypes, but compared to WT, this shift was reduced in β_1_-tg, while more pronounced in β_1_-tg/Gα_i2_^−/−^. In contrast to Gα_i3_, Gα_i2_ deficiency was associated with significant effects on the inactivation rates of *I*_CaL_ in β_1_-tg myocytes, as reflected by delayed inactivation over almost the entire voltage range (Fig. S3). Even at 4–5 months of age, the absence of Gα_i3_ (β_1_-tg/Gα_i3_^−/−^) in contrast to Gα_i2_ (β_1_-tg/Gα_i2_^−/−^) appeared to shift basal *I*_CaL_ properties towards WT levels, while as in β_1_-tg mice, the response to β-adrenergic stimulation was blunted. However, here, data are limited to five recordings under each condition with myocytes from a single animal.

In summary, we found that in contrast to Gα_i3_, the lack of Gα_i2_ does not normalize basal *I*_CaL_ in β_1_-tg mice, while it restores or even enhances the response to β-adrenergic stimulation.

## Discussion

Cardiac β_1_-AR overexpression leads to heart failure in mice (Engelhardt et al. 1999; Lee et al. 2015; Schröper et al. 2024). Additional Gα_i3_ deficiency was protective (Schröper et al. 2024), while Gα_i2_ deficiency exacerbated cardiomyopathy in both β_1_- and β_2_-AR overexpressing mice (Foerster et al. 2003; Keller et al. 2015). In β_2_-tg mice, ventricular *I*_CaL_ was reduced (Heubach et al. 2001; Foerster et al. 2003, 2004), perhaps due to increased ventricular Gα_i3_ expression (Foerster et al. 2003; Dizayee et al. 2011), suggested by enhanced LTCC activity when lacking Gα_i3_ (Klein 2009). Consistently, we find Gα_i3_ deficiency to revert the reduction in basal *I*_CaL_ density in β_1_-tg mice. This was not the case in Gα_i2_-deficient β_1_-tg mice. Non-selective G_i_-protein inhibition restored the reduced contractile response to β-adrenergic stimulation in failing cardiomyocytes (Brown and Harding 1992). Thus, the blunted LTCC response to β-adrenergic stimulation in β_1_- and β_2_-tg mice (this study and Foerster et al. 2004) might be explained by G_i_-protein activity. In heart failure, the (PKA-mediated) response to enhanced β-AR stimulation is detrimental in the long term, and β-AR antagonists can decrease patients’ mortality (El-Armouche and Eschenhagen 2009; Baker 2014; Kotecha et al. 2017). Thus, it seems reasonable to consider suppressed β-adrenergic response as protective. We find reduced *I*_CaL_ response on isoproterenol in β_1_-tg mice with and without Gα_i3_ expression. The latter suggests that the above-mentioned restoration of contractile response to β-AR stimulation was not mediated by inhibiting the Gα_i3_ isoform. In contrast, our data on Gα_i2_ deficiency suggest that preventing effects mediated by this isoform may restore or even enhance the response to β-AR stimulation, which could be detrimental in the long term. The observed effects might be explained not only by the lack of a respective Gα_i_ isoform, but upregulation of the other (Dizayee et al. 2011; Köhler et al. 2014; but: Gohla et al. 2007; Hippe et al. 2013). Our recent studies do not support this reactive change of expression, but we might have missed rather slight alterations (Keller et al. 2015; Schröper et al. 2024). Similarly, different relative expression levels of β_1_- and β_2_-AR might play a role, although a previous study suggests the amount of β_2_-AR negligible in β_1_-tg mice (Keller et al. 2015).

In summary, basal *I*_CaL_ and its response to β-adrenergic stimulation is altered in ventricular myocytes from mice overexpressing the cardiac β_1_-AR. Lack of either Gα_i2_ or Gα_i3_ shows differential effects on these alterations.

### The role of ventricular I_CaL_ in the development and prevention of cardiomyopathy

We used ventricular myocytes of mice at an age apparently preceding the onset of ventricular dysfunction or even an effect on survival for two reasons (Keller et al. 2015; Schröper et al. 2024): first, alterations in ventricular LTCC expression and/or function are already found in compensated hypertrophy (Mukherjee and Spinale 1998; Richard et al. 1998). Second, genetic alterations of ventricular *I*_CaL_ can lead to cardiac dysfunction, suggesting a causal role of LTCC (Muth et al. 1999; Nakayama et al. 2007; Beetz et al. 2009; Goonasekera et al. 2012).

Increased ventricular *I*_CaL_ was deleterious in some mouse models (Muth et al. 1999; Nakayama et al. 2007; Beetz et al. 2009). Interestingly, heterozygous knockout of cardiac LTCC expression resulted in reduced *I*_CaL_ density but also hypertrophy and heart failure (Goonasekera et al. 2012). Compared to wild-type littermates, ventricular *I*_CaL_ density was reduced in Gα_i2_-deficient mice but increased in Gα_i3_-deficient mice (Dizayee 2011), but no contractile dysfunction was present in either group (Jain et al. 2001; Keller et al. 2015; Schröper et al. 2024). In β_1_-tg mice, Gα_i3_ deficiency was cardioprotective (Schröper et al. 2024) and largely normalized, i.e. increased, ventricular *I*_CaL_ density, whereas Gα_i2_ deficiency, which was detrimental to contractility and survival in β_1_-tg mice (Keller et al. 2015), did not restore basal *I*_CaL_ density. Regarding an impact on contractility, these results suggest that the mechanism underlying LTCC modulation may play a role.

Since mice in the previous studies died without prior signs (Keller et al. 2015; Schröper et al. 2024) and arrhythmias are the most common cause of death in humans, it is tempting to speculate that the risk of arrhythmias is increased in β_1_-tg/Gα_i2_^−/−^ given the increased window current, which has been associated with rhythm disturbances such as early after depolarizations (Benitah et al. 2010).

We cannot exclude altered LTCC expression, although voltage dependence and response to isoproterenol indicate effects independent of this, and previous studies using β_1_-tg, Gα_i2_- and Gα_i3_-deficient mice did not indicate such changes (Foerster et al. 2004; Dizayee et al. 2011).

### The possible relevance of a reduced response of ventricular I_CaL_ to β-adrenergic stimulation

Sustained stimulation or overexpression of β-AR leads to ventricular hypertrophy and eventually to heart failure in rodent models (Gomes et al. 2013). Acute β-AR stimulation leads to increased LTCC activity like that observed in human heart failure (Tsien et al. 1986; Yue et al. 1990; Schröder et al. 1998), and increased LTCC activity can lead to cardiomyopathy and heart failure in mice (Nakayama et al. 2007; Beetz et al. 2009). Given the life-prolonging effect of heart failure treatment with β-AR antagonists, one might speculate that the reduced *I*_CaL_ response to β-adrenergic stimulation observed in human and murine heart failure (Schröder et al. 1998; Muth et al. 1999; Groner et al. 2004; Foerster et al. 2004; Chen et al. 2008; Beetz et al. 2009) is a protective mechanism, albeit insufficient or decompensating in the long term. Thus, protective effects of Gα_i3_ deficiency in β_1_-tg mice might be due to maintained attenuation of *I*_CaL_ response to β-AR stimulation, whereas in contrast, the deleterious effects of Gα_i2_ deficiency might be linked to restoration of the *I*_CaL_ response to β-adrenergic stimulation, i.e. lack of protection against or increased susceptibility to β-adrenergic stimulation. In addition to the increased window currents under basal conditions, *I*_CaL_ inactivation was delayed upon β-AR stimulation in Gα_i2_-deficient β_1_-tg mice compared to WT or mice solely overexpressing the cardiac β_1_-AR. This might as well contribute to arrhythmia as seen for example with mice expressing a mutated LTCC pore (Cheng et al. 2011; Drum et al. 2014). Studies on the modulation of *I*_CaL_ by muscarinic acetylcholine receptors or β-AR suggest an isoform-specific role of Gα_i2_ or Gα_i3_ (Nagata et al. 2000; Foerster et al. 2003; Klein 2009). This might involve differential regulation of PKA activity that is known to regulate *I*_CaL_ activity (Papa et al. 2022). Of note, we recently found G_i_-isoform-specific differences in phosphorylation of the PKA target phospholamban (Schröper et al. 2024).

### Limitations

Our study is subject to certain limitations. The age of mice used in our experiments was chosen with respect to *in vivo* findings from earlier studies (Keller et al. 2015; Schröper et al. 2024). We cannot exclude that cardiac function is affected already at the age of mice we used now. Again considering previous findings, we used mice at different ages for experiments on either Gα_i2_ or Gα_i3_ deficiency. Of note, *I*_CaL_ alterations in β_1_-tg mice were similar at either age, and differences between β_1_-tg mice lacking either Gα_i2_ or Gα_i3_ were similar at 4–5 months of age, though indicated by experiments with myocytes from only one β_1_-tg/Gα_i3_^−/−^ animal. Experiments with animals of the respective other age are necessary to confirm the hints we found. Since we used exclusively male mice, experiments should be repeated with females. We discuss previous findings obtained with the same mouse lines. Properties of mouse models can change over time. However, not least for animal welfare reasons, it is difficult to repeat experiments. On the other hand, repeating some experiments with animals of the same genotype but a different age seems reasonable. We do not provide sufficient data on molecular mechanisms underlying our findings. Thus, future studies are needed to address the issues discussed and furthermore analyse interaction partners involved in, e.g., adrenergic signalling.

### Summary and conclusion

Given the limitations of your study, we conclude with caution. We assume that Gα_i3_ deficiency contributes to the restoration of contractility in β_1_-tg mice by restoring basal ventricular *I*_CaL_, whereas maintained attenuation of *I*_CaL_ response to β-adrenergic stimulation protects against deleterious effects of enhanced β-AR signalling. In contrast, restored or even enhanced *I*_CaL_ response to β-AR stimulation might explain detrimental effects of Gα_i2_ deficiency observed in β_1_-tg mice previously (Keller et al. 2015). Of course, other factors besides *I*_CaL_ may be relevant for the effects of Gα_i2_ or Gα_i3_ deficiency. Overall, our current and previous data suggest that isoform-specific effects of inhibitory G proteins should be further explored regarding new options for treatment or prevention of heart failure.

## Supplementary Information

Below is the link to the electronic supplementary material.

Supplementary file1 (JPG 276 KB)

Supplementary file2 (JPG 265 KB)

Supplementary file3 (JPG 71 KB)

Supplementary file4 (DOCX 16.7 KB)

Supplementary file5 (DOCX 16.8 KB)

Supplementary file6 (DOCX 72.5 KB)

## Data Availability

All source data for this work (or generated in this study) are available upon reasonable request.
