# Supplementary figures and images for: Opposite effects of Gαi2 or Gαi3 deficiency on reduced basal density and attenuated β-adrenergic response of ventricular Ca2+ currents in myocytes of mice overexpressing the cardiac β1-adrenoceptor
Source: Naunyn Schmiedebergs Arch Pharmacol. 2025 Mar 31;398(9):12543–9. doi: 10.1007/s00210-025-03999-y (PMC12449356; doi:10.1007/s00210-025-03999-y)

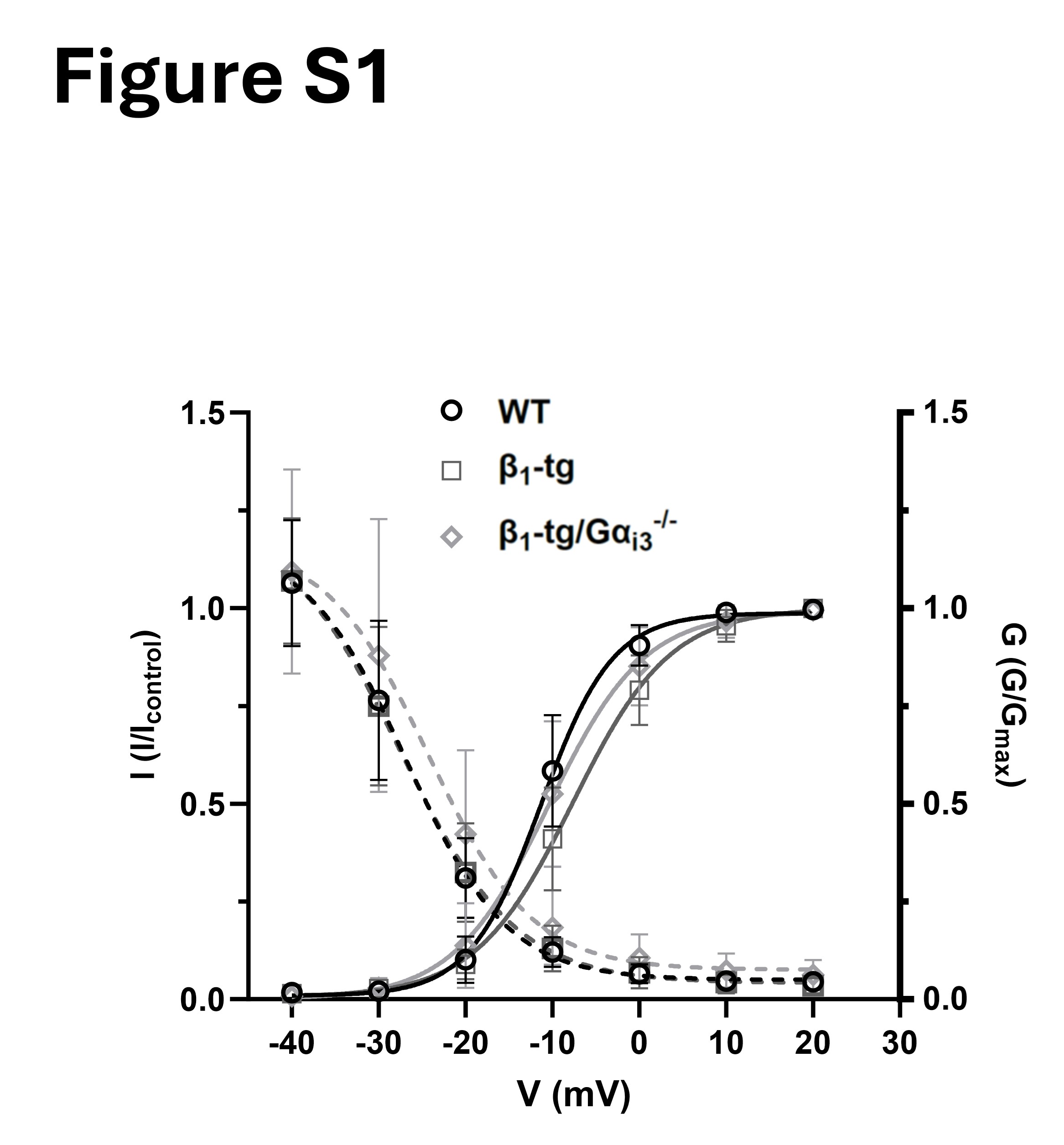

Supplement: Supplementary file 1 — Supplementary file1 (JPG 276 KB) [file 210_2025_3999_MOESM1_ESM.jpg]

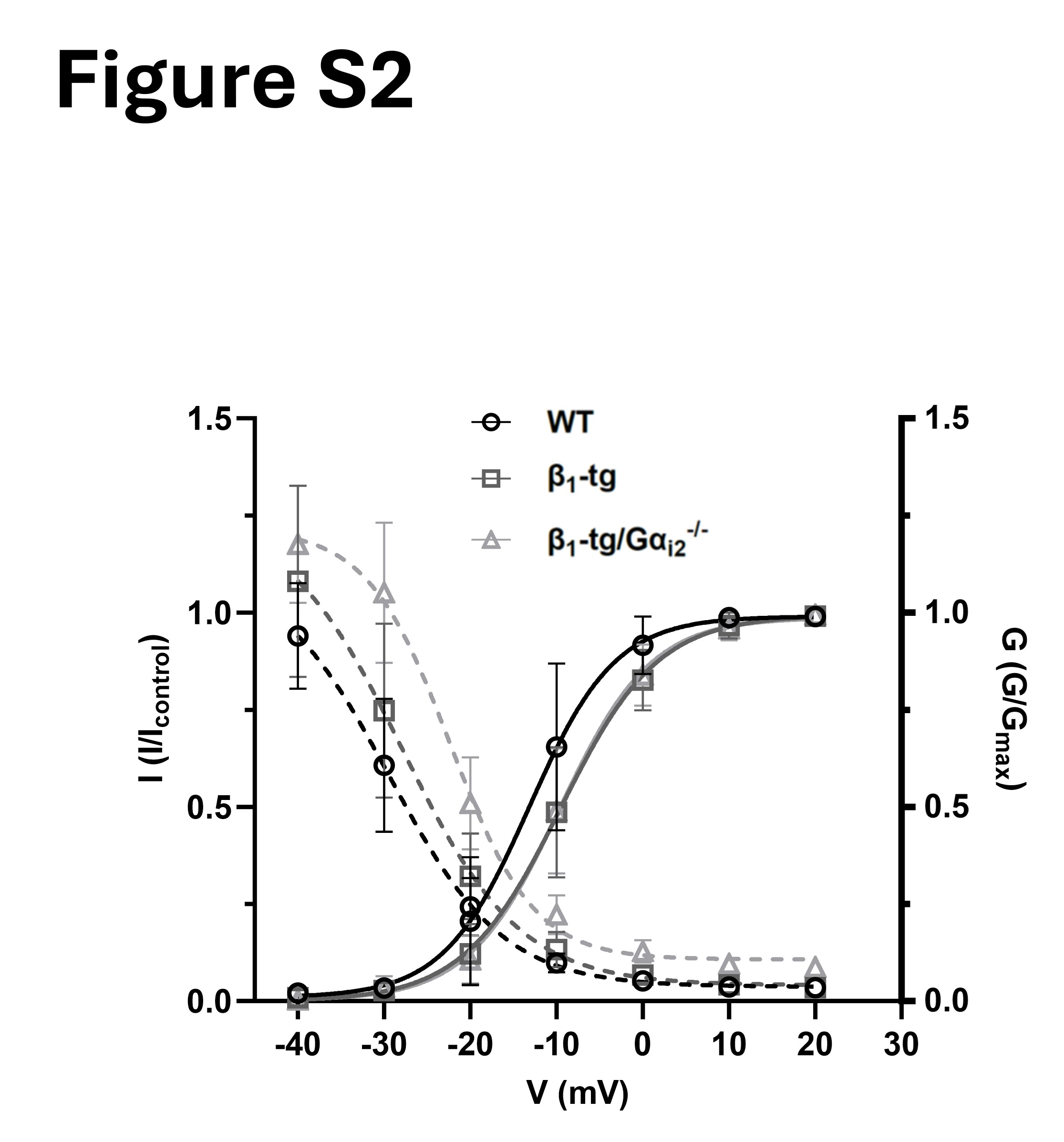

Supplement: Supplementary file 2 — Supplementary file2 (JPG 265 KB) [file 210_2025_3999_MOESM2_ESM.jpg]

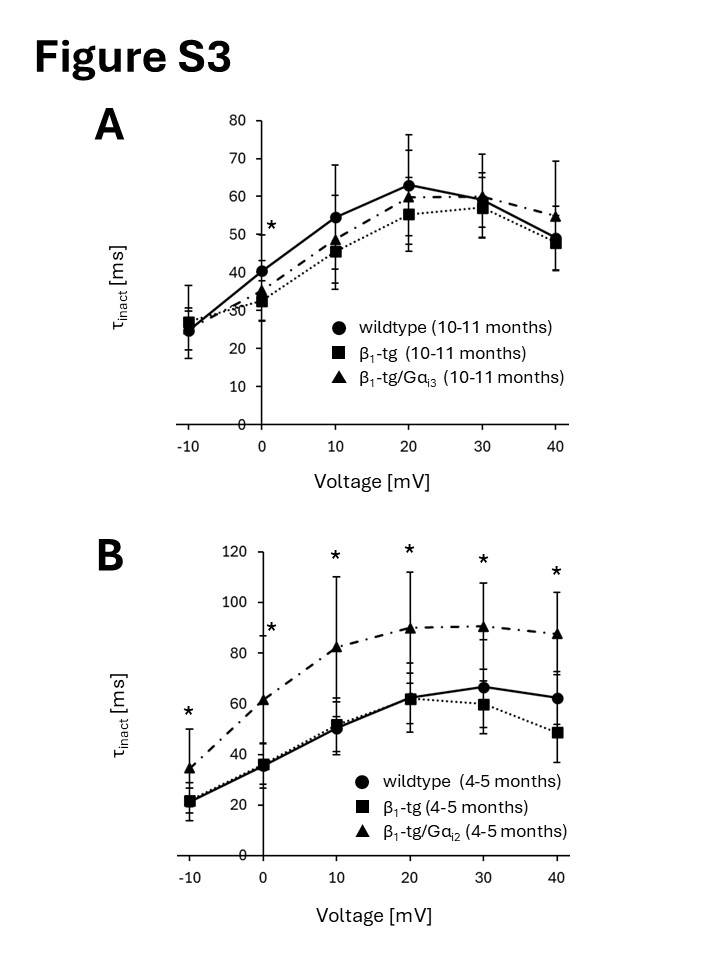

Supplement: Supplementary file 3 — Supplementary file3 (JPG 71 KB) [file 210_2025_3999_MOESM3_ESM.jpg]
