# Supplementary material for: Opposite effects of Gαi2 or Gαi3 deficiency on reduced basal density and attenuated β-adrenergic response of ventricular Ca2+ currents in myocytes of mice overexpressing the cardiac β1-adrenoceptor
Source: Naunyn Schmiedebergs Arch Pharmacol. 2025 Mar 31;398(9):12543–9. doi: 10.1007/s00210-025-03999-y (PMC12449356; doi:10.1007/s00210-025-03999-y)
Supplement: Supplementary file 4 — Supplementary file4 (DOCX 16.7 KB) [file 210_2025_3999_MOESM4_ESM.docx]

**Table S1****: Effect of Gα_i3_ deficiency on ventricular I_CaL_ in β_1_-tg mice aged 10-11 months.** Peak I_CaL_ density and half-maximum potentials of activation (V_0.5_act_) and inactivation (V_0.5_inact_) obtained with ventricular myocytes isolated from wildtype mice, mice overexpressing the cardiac β_1_-adrenoceptor (β_1_-tg) and β_1_-tg mice globally lacking Gα_i3_ (β_1_-tg/Gα_i3_^-/-^). At least three mice aged 10-11 months were examined per genotype. Patch-clamp recordings were performed with different sets of cells either under basal conditions or after incubation with 1 µM isoproterenol (iso) for 8 ± 2 minutes. Data are given as mean ± SD. Number of underlying recordings are given in brackets. Asterisks indicate p values obtained from unpaired t tests used to analyze iso effects or from comparison of genotypes under basal conditions using Bonferroni-corrected post-tests following one-way ANOVA (*: < 0.05; **: < 0.01; ***: < 0.001). ns: p > 0.05.

| **parameter** | **wildtype** | | **β_1_-tg** | | **β_1_-tg/Gα_i3_^-/-^** | | **p values (basal)** | | |
| --- | --- | --- | --- | --- | --- | --- | --- | --- | --- |
|  | **basal** | **+ iso** | **basal** | **+ iso** | **basal** | **+ iso** | **WT vs.**  **β_1_-tg** | **WT vs.**  **β_1_-tg/Gα_i3_^-/-^** | **β_1_-tg vs.**  **β_1_-tg/Gα_i3_^-/-^** |
| peak I_CaL_ [pA/pF] | -8.1 ± 1.6 (18) | -13.6 ± 5.2** (12) | -5.5 ± 1.6 (17) | -7.4 ± 1.9** (12) | -7.5 ± 1.6 (19) | -9.5 ± 3.6* (18) | *** | ns | ** |
| V_0.5_act_ [mV] | -11.3 ± 2.5 (18) | -17.0 ± 4.0*** (12) | -7.7 ± 2.8 (17) | -9.7 ± 4.6 (12) | -10.6 ± 4.3 (19) | -14.2 ± 6.7 (18) | ** | ns | * |
| V_0.5_inact_ [mV] | -26.0 ± 3.1 (15) | -29.5 ± 4.4* (14) | -26.0 ± 3.2 (16) | -26.9 ± 4.5 (14) | -24.7 ± 6.4 (16) | -29.9 ± 4.8** (12) | ns | ns | ns |
