# Supplementary material for: Opposite effects of Gαi2 or Gαi3 deficiency on reduced basal density and attenuated β-adrenergic response of ventricular Ca2+ currents in myocytes of mice overexpressing the cardiac β1-adrenoceptor
Source: Naunyn Schmiedebergs Arch Pharmacol. 2025 Mar 31;398(9):12543–9. doi: 10.1007/s00210-025-03999-y (PMC12449356; doi:10.1007/s00210-025-03999-y)
Supplement: Supplementary file 5 — Supplementary file5 (DOCX 16.8 KB) [file 210_2025_3999_MOESM5_ESM.docx]

**Table S2:** **Effect of Gα_i2_ deficiency on ventricular I_CaL_ in β_1_-tg mice aged 4-5 months.** Peak I_CaL_ density and half-maximum potential of activation (V_0.5_act_) and inactivation (V_0.5_inact_) obtained with ventricular myocytes isolated from wildtype mice, mice overexpressing the cardiac β_1_-adrenoceptor (β_1_-tg) and β_1_-tg mice globally lacking Gα_i2_ (β_1_-tg/Gα_i2_^-/-^). At least three mice aged 4-5 months were examined per genotype. Patch-clamp recordings were performed with different sets of cells either under basal conditions or after incubation with 1 µM isoproterenol (iso) for 8 ± 2 minutes. Data are given as mean ± SD. Number of underlying recordings are given in brackets. Asterisks indicate p values obtained from unpaired t tests used to analyze iso effects or from comparison of genotypes under basal conditions using Bonferroni-corrected post-tests following one-way ANOVA (*: < 0.05; **: < 0.01; ***: < 0.001). ns: p > 0.05.

| **parameter** | **wildtype** | | **β_1_-tg** | | **β_1_-tg/Gα_i2_^-/-^** | | **p values (basal)** | | |
| --- | --- | --- | --- | --- | --- | --- | --- | --- | --- |
|  | **basal** | **+ iso** | **basal** | **+ iso** | **basal** | **+ iso** | **WT vs.**  **β_1_-tg** | **WT vs.**  **β_1_-tg/Gα_i2_^-/-^** | **β_1_-tg vs.**  **β_1_-tg/Gα_i2_^-/-^** |
| peak I_CaL_ [pA/pF] | -8.1 ± 1.9 (15) | -12.4 ± 2.7*** (7) | -5.7 ± 1.8 (11) | -7.9 ± 2.7* (9) | -5.8 ± 2.1 (17) | -12.2 ± 2.9*** (9) | * | ** | ns |
| V_0.5_act_ [mV] | -13.3 ± 4.7 (15) | -20.4 ± 3.4** (7) | -9.4 ± 3.6 (11) | -14.5 ± 4.5* (9) | -9.6 ± 3.1 (17) | -22.1 ± 4.6*** (9) | ns | * | ns |
| V_0.5_inact_ [mV] | -28.8 ± 3.7 (10) | -28.1 ± 2.0 (5) | -26.4 ± 3.6 (14) | -25.6 ± 3.1 (9) | -21.4 ± 1.7 (13) | -19.8 ± 2.6 (8) | ns | *** | *** |
