# Supplementary material for: Opposite effects of Gαi2 or Gαi3 deficiency on reduced basal density and attenuated β-adrenergic response of ventricular Ca2+ currents in myocytes of mice overexpressing the cardiac β1-adrenoceptor
Source: Naunyn Schmiedebergs Arch Pharmacol. 2025 Mar 31;398(9):12543–9. doi: 10.1007/s00210-025-03999-y (PMC12449356; doi:10.1007/s00210-025-03999-y)
Supplement: Supplementary file 6 — Supplementary file6 (DOCX 72.5 KB) [file 210_2025_3999_MOESM6_ESM.docx]

**Material and methods**

**Mouse models**

We had backcrossed mice with cardiac overexpression of the human β_1_-AR (β_1_-tg) from an FVB/N background (TG4 in (Engelhardt et al., 1999)) to a C57BL/6J background for an earlier study (Keller et al., 2015). This allowed for crossbreeding with mice globally lacking Gα_i2_ (Dizayee et al., 2011; Foerster et al., 2003) or Gα_i3_ (Gohla et al., 2007), to produce β_1_‑tg with the respective isoform-specific Gα_i_ deficiency (β_1_‑tg/Gα_i2_^‑/‑^ and β_1_‑tg/Gα_i3_^‑/‑^, respectively). Age-matched wildtype littermates served as controls. Housing was performed in individually ventilated cages with a 12h/12h dark/light cycle and food and water ad libitum. For genotyping, ear clips from three-week-old mice were processed to obtain genomic DNA that was used in genotyping PCR for Gα_i2_, Gα_i3_ and the β_1_-AR as described previously (Dizayee et al., 2011; Keller et al., 2015). Animals were killed by cervical dislocation. Only male mice were used. Since we were interested in putative effects preceding and perhaps causing cardiomyopathy and heart failure, we used mice at an age of about 10-11 months for investigation of the role of Gα_i3_. At this age neither β_1_-tg nor β_1_-tg/Gα_i3_^-/-^ mice displayed ventricular hypertrophy or dysfunction in our recent study (Schröper et al., 2024). Since β_1_-tg/Gα_i2_^-/-^ already showed significant ventricular dysfunction and increased mortality at an age around 10-11 months , in the current study we analysed β_1_-tg/Gα_i2_^-/-^ at 4-5 months of age, i. e. shortly before the survival curves began to diverge (Keller et al., 2015). For both analyses, age-matched wildtype and β_1_-tg mice were used for comparison. The responsible federal state authority approved animal breeding, maintenance and experiments (Landesamt fuer Natur-, Umwelt- und Verbraucherschutz Nordrhein-Westfalen; references: 84-02.04.2016.A422 and 81-02.04.2022.A141). All animal experiments complied with the guidelines from Directive 2010/63/EU of the European Parliament on the protection of animals used for scientific purposes.

**Myocyte isolation**

Ventricular myocytes were isolated from male mice (n=3-6 per group). 30-40 min after i.p. injection of heparin (500 IU/mL per g body weight) mice were killed by cervical dislocation. Hearts were removed, prepared in cold Ca^2+^-free perfusion buffer (in mM: 5.5 glucose, 10 2,3-Butanedione monoxime, 10 HEPES, 30 taurine, 113 NaCl, 4.7 KCl, 0.6 KH_2_PO_4,_ 0.6 (Na_2_HPO_4_)_2_H_2_O, 1.2 MgSO_4_-7H_2_O, 12 NaHCO_3,_ 10 KHCO_3_ adjusted to pH 7.46 with NaOH) and cannulated via the aorta. Immediately after cannulation, hearts were subjected to retrograde perfusion at 37°C. After 6-8 min washing with perfusion buffer at a flow rate of 3 mL/min, perfusion was switched to digestion buffer containing 300 U/mL collagenase II (CLS-2; Worthington), 0.04 mg/mL protease and 12.5 µM CaCl_2_ for 9-10 min. After removing the atria and big vessels, ventricles were minced with fine forceps and the tissue was gently agitated with a Pasteur pipette to dissociate the cells. Perfusion buffer with additional 10% FCS and 12.5 μM CaCl_2_ was added at room temperature to stop digestion, and the resulting suspension was filtered through a 250 µm polyamide mesh before cells were allowed to sediment for 20 min. Supernatant was discarded and procedure was repeated, gradually increasing the Ca^2+^ concentration up to 1.2 mM. Cells were kept at room temperature and subjected to patch-clamp experiments within 2-8 hours after isolation.

**Electrophysiological studies**

Using the whole-cell configuration of the patch-clamp technique, ventricular L-type Ca^2+^ currents (I_CaL_) were recorded (amplifier: Axopatch 200B; A/D converter: Digidata 1440A; Axon Instruments, Sunnyvale, CA, USA). Pipette solution (mM): 120 CsCl, 10 EGTA, 4 Mg-ATP, 5 HEPES, 1 MgCl_2_; pH 7.2. Bath solution (mM): 137 NaCl, 10 HEPES, 10 Glucose, 5.4 CsCl, 2 CaCl_2_, 1 MgCl_2_; pH 7.4. To obtain I-V curves, I_CaL_ was recorded at room temperature using a double-pulse protocol (i.e. 45 ms pre-pulse from a holding potential of -80 mV to -40 mV to inactivate Na^+^ channels and T-type Ca^2+^ channels, followed by 150 ms test pulses from -40 mV to +50 mV in 10 mV increments). To correct for differences in cell size, I_CaL_ density was used for analysis, i.e. peak I_CaL_ at a given test potential divided by the cell’s membrane capacitance. Cells’ membrane capacitance was similar with 209 ± 43 (wildtype at 4-5 months), 212 ± 57 (β_1_-tg at 4-5 months of age), 213 ± 69 (β_1_-tg/Gα_i2_^-/-^), 223 ± 15 (wildtype at 10-11 months), 220 ± 60 (β_1_-tg at 10-11 months of age), and 200 ± 44 pF (β_1_-tg/Gα_i3_^-/-^ ). For analysing voltage dependence of activation, data were fitted by combined Ohm and Boltzmann relation using the equation $I(V) = (V - VR) x\frac{G_{max}}{(1+\exp\frac{(V_{0.5}-V)}{dV})}$ (Despang et al., 2022; Dizayee et al., 2011). We estimated half-maximum potential of inactivation (V_0.5 inact_) from steady-state inactivation (SSI) curves by fitting with a sigmoidal Boltzmann equation, too. In brief, SSI was obtained by a double-pulse protocol using voltage steps from -60 to +60 mV with pulse intervals of 10 seconds (Poomvanicha et al., 2011). In addition to the measurements of I_CaL_ under basal conditions, patch-clamp recordings were performed in a separate set of experiments with cells incubated with 1 µM isoproterenol (iso) for 8 to 10 minutes. For a 1 mM stock solution, iso was dissolved in H_2_O together with ascorbic acid (0.1%), further dissolved to 0.1 mM iso, and then stored in aliquots at +4° C for 6 weeks maximum. For experiments, 30 µL of the 0.1 mM iso solution was added to the 3 mL bath solution used for electrophysiological recordings.

**Statistics**

Throughout, we present mean values ± standard deviation (SD). When comparing more than two groups, we applied one-way ANOVA followed by Bonferroni-corrected post-tests. For comparison of only two groups, we used an unpaired Student’s t test or the Mann-Whitney U test as appropriate. We considered a p-value < 0.05 statistically significant. Of note, we performed our statistical analyses with an exploratory intention. For the sake of readability, we use asterisks to indicate p-values in figures and tables as indicated in the respective legends. For statistical analyses and creation of Figures we used GraphPad Prism, Excel and PowerPoint.

**References**

Despang, P., Salamon, S., Breitenkamp, A., Kuzmenkina, E., & Matthes, J. (2022). Inhibitory effects on L- and N-type calcium channels by a novel CaVβ1 variant identified in a patient with autism spectrum disorder. *Naunyn-Schmiedeberg’s Archives of Pharmacology*, *395*(4), 459–470. https://doi.org/10.1007/S00210-022-02213-7

Dizayee, S., Kaestner, S., Kuck, F., Hein, P., Klein, C., Piekorz, R. P., Meszaros, J., Matthes, J., Nürnberg, B., & Herzig, S. (2011). Gαi2- and Gαi3-Specific Regulation of Voltage-Dependent L-Type Calcium Channels in Cardiomyocytes. *PLoS ONE*, *6*(9), e24979. https://doi.org/10.1371/journal.pone.0024979

Engelhardt, S., Hein, L., Wiesmann, F., & Lohse, M. J. (1999). Progressive hypertrophy and heart failure in β1-adrenergic receptor transgenic mice. *Proceedings of the National Academy of Sciences of the United States of America*, *96*(12), 7059–7064. https://doi.org/10.1073/pnas.96.12.7059

Foerster, K., Groner, F., Matthes, J., Koch, W. J., Birnbaumer, L., & Herzig, S. (2003). Cardioprotection specific for the G protein Gi2 in chronic adrenergic signaling through 2-adrenoceptors. *Proceedings of the National Academy of Sciences*, *100*(24), 14475–14480. https://doi.org/10.1073/pnas.1936026100

Gohla, A., Klement, K., Piekorz, R. P., Pexa, K., Dahl, S., Spicher, K., Dreval, V., & Ha, D. (2007). An obligatory requirement for the heterotrimeric G protein G i3 in the antiautophagic action of insulin in the liver. *Proceedings of the National Academy of Sciences*, *104*(8), 3003–3008.

Keller, K., Maass, M., Dizayee, S., Leiss, V., Annala, S., Köth, J., Seemann, W. K., Müller-Ehmsen, J., Mohr, K., Nürnberg, B., Engelhardt, S., Herzig, S., Birnbaumer, L., & Matthes, J. (2015). Lack of Gαi2 leads to dilative cardiomyopathy and increased mortality in β1-adrenoceptor overexpressing mice. *Cardiovascular Research*, *108*(3), 348–356. https://doi.org/10.1093/cvr/cvv235

Poomvanicha, M., Wegener, W., Blaich, A., Fischer, S., Domes, K., & Moosmang, S. (2011). *Facilitation and Ca 2 ؉ -dependent Inactivation Are Modified by Mutation of the Ca v 1 . 2 Channel IQ Motif **. *286*(30), 26702–26707. https://doi.org/10.1074/jbc.M111.247841

Schröper, T., Mehrkens, D., Leiss, V., Tellkamp, F., Engelhardt, S., Herzig, S., Birnbaumer, L., Nürnberg, B., & Matthes, J. (2024). Protective effects of Gαi3 deficiency in a murine heart-failure model of β1-adrenoceptor overexpression. *Naunyn-Schmiedeberg’s Archives of Pharmacology*, *397*(4), 2401–2420. https://doi.org/10.1007/s00210-023-02751-8
